# Silicon Supplementation Modulates Physiochemical Characteristics to Balance and Ameliorate Salinity Stress in Mung Bean

**DOI:** 10.3389/fpls.2022.810991

**Published:** 2022-05-18

**Authors:** Musa Al Murad, Sowbiya Muneer

**Affiliations:** ^1^Horticulture and Molecular Physiology Lab, School of Agricultural Innovations and Advanced Learning, Vellore Institute of Technology, Vellore, India; ^2^School of Biosciences and Technology, Vellore Institute of Technology, Vellore, India

**Keywords:** amelioration, physiochemical characteristics, mung bean, salinity stress, silicon

## Abstract

Mung bean is a low-cost high-protein legume that is sensitive to salinity. Salt stress has been demonstrated to be mitigated by silicon (Si). In legumes, the potential for silicon (Si)-mediated abiotic stress reduction has mainly been ignored. Moreover, there is little information on the specific role of comparable Si (sodium silicate) concentrations in salinity stress reduction. As a result, the current study investigated the impact of two distinct Si concentrations (1 and 5 mM) on the physiochemical features of the “mung bean,” one of the most extensively cultivated legumes, when exposed to salinity (10, 20, and 50 mM NaCl). Salinity stress reduced growth variables such as biomass, nodule formation, plant length, height, and photosynthetic measures, which were mitigated by silicon supplementation at 5 mM sodium silicate. The inclusion of silicon increased the expression of photosynthetic proteins such as PSI, PSII, and LHCs under salt stress. Salinity stress also caused oxidative damage in the mung bean in the form of hydrogen peroxide (H_2_O_2_) and superoxide radical (O_2_^−^), leading in increased lipid peroxidation (MDA) and electrolyte leakage. In contrast, 5 mM sodium silicate tends to scavenge free radicals, reducing lipid peroxidation (MDA) and electrolyte loss. This was linked to significant silica deposition in the leaf epidermis, which eventually functioned as a mechanical barrier in mitigating the deleterious effects of salt stress. Si supplementation also decreased Na^+^ uptake while increasing K^+^ uptake. Silicon, specifically 5 mM sodium silicate, was found to minimize salinity stress in mung bean by altering physio-chemical parameters such as photosynthetic machinery, Na^+^/K^+^ homeostasis, mechanical barriers, osmolyte production, and oxidative stress.

## Introduction

Salinity is one of the major abiotic stresses that has reportedly affected around 20% of the irrigated land accounting to 60 million hectares worldwide, thus arising concerns about its negative ecological and socio-economic outcomes (Munns and Tester, 2008; Islam et al., 2016; Sehrawat et al., 2018). This problem is expected to worsen in the coming years, due to continued global warming, extreme climate fluctuations, and unmonitored use of fertilizers, industrial pollution, and irrigation with saline water (Zhu et al., 2019; Meng et al., 2020).

Salinity stress inhibits plant growth and development by decreasing osmotic potential in soil solutions and increasing ion toxicity, particularly of Na^+^ and Cl^−^ ions (Ashraf and Akram, 2009; Daoud et al., 2018). The buildup of Na^+^ and Cl^−^ at exceptionally high levels during salinity stress largely impedes plant nutrient uptake (Ahanger and Agarwal, 2017). Salinity stress also causes a decrease in the Calvin cycle, a decrease in CO_2_ intake, and a decrease in photosynthetic pigments such as chlorophyll concentration, all of which have an effect on the net photosynthesis rate (Sairam et al., 2002; Munns et al., 2020). By increasing the Na^+^/K^+^ ratio, salinity stress slows various other metabolic processes in crops, including transpiration, stomatal conductance, and PSII quantum yield (Gupta and Huang, 2014). In fact, photoinhibition caused by salinity stress results in the accumulation of untapped energy, which destroys the photosynthetic apparatus, leading in the production of reactive oxygen species (ROS; Nishiyama and Murata, 2014; Gururani et al., 2015; Tokarz et al., 2021). Excessive ROS concentrations in cells weaken the antioxidant apparatus, resulting in lipid peroxidation (MDA), which affects the cell membrane structure and permeability (Davey et al., 2005) and also damages lipids, proteins, and nucleic acid (Tanveer, 2020). As a result, the worrying biochemical and physiological changes that result in diminished plant growth and development might be related to salinity stress.

Mung bean (*Vigna radiata* L.) is an important legume crop that supplies a significant quantity of protein (24.5%), carbs (59.9%), fatty acids (1.2%), and fibers (4.5%), as well as vital amino acids (Fuller, 2007; Khattak et al., 2008; Aasim et al., 2019). Around 3 million tons of this economic crop are produced annually, accounting for 5% of the total pulse production worldwide (World Vegetable Centre, 2018). Due to its high nutritional value and global production, mung bean remains a staple source of protein for millions of people in developing countries (Sehrawat et al., 2013). However, legumes such as mung bean are cultivated in arid and semi-arid regions which are prone to salt stress and hence are considered salt-sensitive crops (Sprent and Gehlot, 2010). It has been reported that salinity limits the yield of mung bean to almost 70% at 50 mM NaCl and exerts a negative effect on the quality of the crop (Saha et al., 2010; Sehrawat et al., 2013). Besides, salinity also affects its seed germination, seedling development, radicle length (Dutta and Bera, 2014; Sehrawat et al., 2014), whole plant growth, biomass (Ahmed, 2009), nutrient accumulation (Naher and Alam, 2010), photosynthesis (Koyro, 2006), ROS formation, relative water content, membrane stability index, chlorophyll and carotenoid content (Sehrawat et al., 2015), nodulation, nodule respiration, and root hair formation (Swaraj and Bishnoi, 1999; Naher and Alam, 2010). As a result, there is an urgent need to develop a robust and long-term strategy for conferring salt tolerance on the mung bean, which is an important leguminous and food crop.

The upgradation of silicon (Si) from a “nonessential” substance to the status of a “beneficial substance” by the International Nutrition Institute (IPNI) in 2015 has established the fact that Si is not directly involved in plant growth, function or metabolic processes, but ameliorates the negative effects of biotic and abiotic stresses, and in turn reflects on the improvements in plant growth, function, and metabolic processes (Gao et al., 2011; Coskun et al., 2018; Flam-Shepherd et al., 2018). For example, several studies have shown Si-mediated alleviation of salinity stress in crops like capsicum (Manivannan et al., 2016; Abdelaal et al., 2020), tomato (Muneer and Jeong, 2015; Hoffmann et al., 2020), sorghum (Liu et al., 2015), okra (Abbas et al., 2015), cucumber (Zhu et al., 2004; Yin et al., 2019), wheat (Alzahrani et al., 2018; Javaid et al., 2019), rice (Shi et al., 2013), basil (Farouk et al., 2020), alfalfa (Meng et al., 2020), and soybean (Chung et al., 2019). The mechanisms by which Si infers salinity stress tolerance to plants include: (a) retention of water content at optimal levels (Zhu and Gong, 2014), (b) improving physiological attributes such as leaf area expansion, light interception leading to increased photosynthetic rates (Ma et al., 2006), (c) limiting the oxidative stress by regulating ion toxicity, (d) regulation of compatible solutes such as betaine, glycine, and proline which stabilize important membrane proteins (Etesami and Jeong, 2018), (e) accumulation of polyamines which prevent Na^+^ and K^+^ flow *via* non-selective cationic channels (Zhao et al., 2007), and (f) increasing performance of antioxidant enzymes and efficiency of PSII (Al-Aghabary et al., 2004).

Despite the fact that various studies on Si-mediated stress mitigation in plants have been conducted, the effect of Si supplementation in legumes has mainly been overlooked. The Poaceae family receives the majority of silicon research (42%), whereas the Fabaceae family receives only 7% (Putra et al., 2021), emphasizing the urgent need to enhance the focus of Si research on legumes as well. Also, no report on a comparison analysis of two distinct concentrations of the same source of Si (sodium silicate) in any crop/plant has been published to date. Variable impacts of Si sources have been reported among plant species. However, it would be fascinating to determine if or not the effects of Si-mediated stress reduction are concentration dependent. As a result, we attempted to evaluate the impacts of two different sodium silicate concentrations on the physiochemical characteristics of mung bean exposed to salt stress in our study. The present study depicted that silicon (sodium silicate) ameliorates and balances the salinity stress by modulating physiochemical characteristics in mung bean. Moreover, a significant physiological mechanism was also inculcated on silicon can alleviate salinity stress in mung bean.

## Materials and Methods

### Plant Materials, Growth Conditions, and Treatments

**Figure 1:**
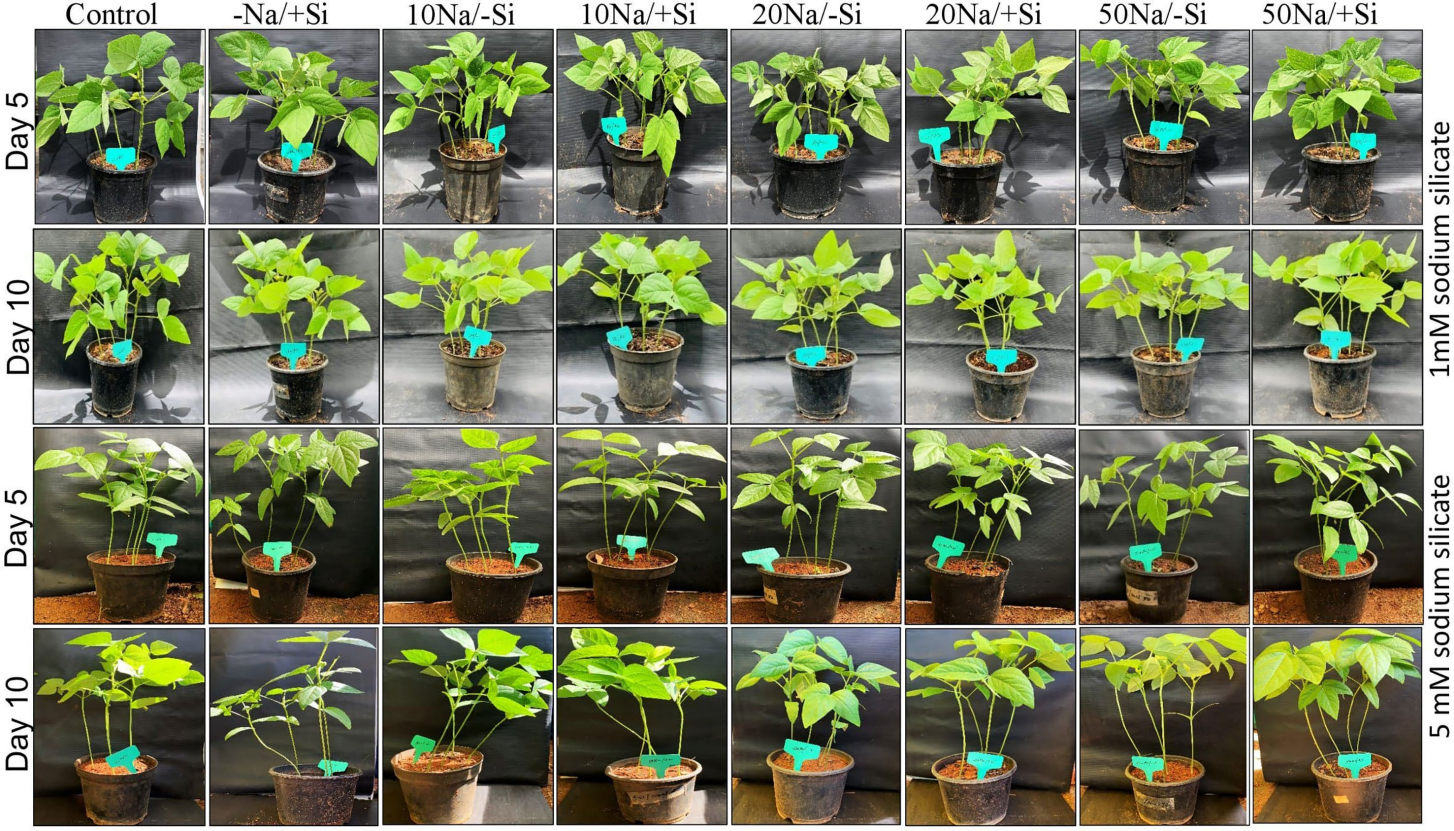
Experimental setup of mung bean (*Vigna radiata*) under 1 mM (Si_1_) and 5 mM (Si_2_) Si supply and salinity stress after eight treatments: (i) control (T1), (ii) −NaCl+Si (1 mM/5 mM) (T2), (iii) 10 mM NaCl/−Si (T3), (iv) 10 mM NaCl/+Si (1 mM/5 mM) (T4), (v) 20 mM NaCl/−Si (T5), (vi) 20 mM NaCl/+Si (1 mM/5 mM) (T6), (vii) 50 mM NaCl/−Si (T7), and (viii) 50 mM NaCl/+Si (1 mM/5 mM) (T8) for a period of 10 days.

Seeds of mung bean (*Vigna radiata* L.) collected from local growers were surface-sterilized with 5% (*v*/*v*) sodium hypochlorite solution for 30 min and further washed with distilled water. The seeds were then sown in pots filled with sterilized red soil, sand, and vermicompost in the ratio 1:1:1 and grown in polyhouse of VIT School of Agricultural Innovations and Advanced Learning (VAIAL), VIT, Vellore, India. The pots had a diameter and height of 13 and 17 cm, respectively. Four seedlings were sown in each pot. The temperature of the greenhouse was maintained between 27 and 30°C with a relative humidity of 60–70% (monitored with the help of digital temperature sensor; Richie HTC infrared/Optical thermometer). After 30 days, the pots were divided randomly for salinity and silicon treatments. For silicon treatment, two concentrations of sodium silicate were used in the experiment, *viz.* 1 mM (Si_1_) and 5 mM (Si_2_). Our experiment was comprised of the following eight different treatments: (i) control (T1) (ii) −NaCl+Si (1 mM/5 mM) (T2), (iii) 10 mM NaCl/−Si (T3), (iv) 10 mM NaCl/+ Si (1 mM/5 mM) (T4), (v) 20 mM NaCl/−Si (T5), (vi) 20 mM NaCl/+Si (1 mM/5 mM) (T6), (vii) 50 mM NaCl/−Si (T7), (viii) 50 mM NaCl/+Si (1 mM/5 mM) (T8) (Figure 1). The experiments were designed in a completely randomized manner with four replicates for each treatment. For silicon treatment, the plants were irrigated with sodium silicate (1 mM/5 mM) solution followed by intermittent irrigation with normal water for the period of 10 days. Leaf samples were collected after 5 and 10 days of treatments, respectively, and stored at −80°C for further experimentation.

### Plant Growth and Biomass Yield

Plants were uprooted from pots, washed with distilled water, and then weighed in a digital weighing balance for fresh biomass measurement. For measuring dry biomass, plant samples were placed in a hot air oven for 48 h at 65–75°C and then weighed subsequently. The lengths of shoot and root were measured using a measuring scale.

### Determination of Gaseous Exchange Parameters and Quantum Yield of PSII

The net photosynthesis rate, stomatal conductance, and transpiration rate were measured using a portable SPAD meter (Konica Minolta, Tokyo, Japan). A mini-PAM 2000 chlorophyll fluorescence meter (Heinz Walz GmbH) was used to measure the chlorophyll fluorescence (*F_v_*/*F_m_*). The leaves were adapted to dark conditions 30 min before measurement. The maximum PSII quantum yield (*F_v_*/*F_m_*) was calculated as *F_v_*/*F_m_* = (*F_m_* − *F*_0_)/*F_m_* (Yang et al., 2013).

### Determination of Pigments

The content of the photosynthetic pigments, *viz.* chlorophyll and carotenoid, was determined according to the method of Hiscox and Israelstam (1979). Briefly, 1 g of leaf samples was immersed in 5 ml of dimethyl sulfoxide (DMSO) and incubated in a hot water bath (65°C) for 1 h for the pigments to leach out. Following incubation, the absorbance of the samples was determined using an UV-VIS spectrophotometer at wavelengths 410, 510, 663, and 445 nm, respectively. The content of chlorophyll and carotenoid was then calculated using the formulae given by Arnon and Israelstam (1949).

### Analysis of Multiprotein Complex Proteins in Thylakoids (1D-BN-PAGE)

Blue native polyacrylamide gel electrophoresis (BN-PAGE) of integral thylakoid proteins was performed according to Muneer et al. (2014) with minor modifications. Two grams of fresh leaf tissues was homogenized in liquid nitrogen, followed by extraction of thylakoid proteins using an extraction buffer (pH 7.8) containing 20 mM Tricine-NaOH, 70 mM sucrose and 5 mM MgCl_2_, which was then filtered through Mira cloth, followed by centrifugation at 4,500 rpm for 10 min. The thylakoid pellets were re-suspended in the above buffer (pH 7.8) and centrifuged again at the same parameters. The pellet containing the thylakoid membrane was then washed with washing buffer (pH 7.0) containing 330 mM sorbitol, 50 mM Bis-Tris-HCl, pH 7.0, and 0.1 mg ml^−1^ pefabloc. An equal volume of re-suspension buffer and solubilization buffer were then added to the pellet, and membrane proteins were allowed to solubilize in ice for 2–3 min. Insoluble material was removed by centrifugation at 18,000 rpm for 15 min. The supernatant was then mixed with 0.1% loading dye (5% CBB-G250, 100 mM Bis-Tris-HCl, pH 7.0, 30% *w*/*v* sucrose, and 500 mM ɛ-amino-*n*-caproic acid) and loaded onto a 5–12.5% acrylamide gradient gel with a constant voltage of 70 V for 15–20 min and followed by 120 V until completion.

### Determination of Relative Water Content (RWC %)

The RWC % was estimated according to Turner and Kramer (1980). Fresh leaves were washed carefully, and their fresh weight (FW) was measured. The leaves were then soaked in distilled water for 4 h at room temperature (25°C) to determine the turgid weight (TW) and further incubated in hot air oven (80°C) for 24 h. The dry weight (DW) of the leaves was then measured. The relative water content (RWC) was measured as follows:


RWC%=(FW−TW)/(DW−TW)×100

where fresh weight (FW); turgid weight (TW); dry weight (DW).

### Determination of Proline Content

Proline content was determined according to Bates et al. (1973). For this, 0.3 g of leaf samples was homogenized in 10 ml of 3% sulfosalicylic acid and was centrifuged at 3,300 rpm for 10 min. Approximately 2 ml of supernatant was taken, to which 2 ml of acid ninhydrin solution and 2 ml of glacial acetic acid were added. The reaction mixture was incubated in hot water bath (100°C) for 1 h and then terminated in ice bath. Further, 4 ml of toluene was added to the mixture with vigorous stirring and the absorbance of the solvent layer was determined at 520 nm.

### Determination of Lipid Peroxidation Level (MDA Content)

The MDA content in leaves was determined according to Heath and Packer (1968). Briefly, 0.5 g of leaf samples was homogenized in 0.1% trichloroacetic acid (TCA) and then centrifuged at 7,000 rpm for 10 min. The supernatant was collected; to it 4 ml of 0.5% thiobarbituric acid (TBA) dissolved in 20% TCA was added. The mixture was then incubated in hot water bath (95°C) for 30 min. Finally, the reaction mixture was placed in ice bath to terminate the reaction. The absorbance was determined at 532 nm and corrected for unspecific turbidity by subtracting the values obtained at 600 nm.

### Determination of Electrolyte Leakage Percentage

The procedure used to determine electrolyte leakage percentage was based on the method of Lutts et al. (1996). Leaf samples were washed with distilled water three times to remove surface contaminants and were then placed in individual stoppered vials containing 10 ml of distilled water. The samples were incubated at room temperature (25°C) on a shaker (100 rpm) for 24 h. After incubation, the electrical conductivity of the bathing solution (EC1) was measured using a conductivity meter (EC tester 11+, Fieldscout, 2265FS). Samples were then incubated in a hot water bath (95°C) for 15 min, and the electrical conductivity (EC2) of the bathing solution was read after cooling it down to the room temperature. ELP was calculated as EC1/EC2 and expressed as %.

### *In situ* Localization of Oxidative Stress Markers (H_2_O_2_ and O_2_^**−**^)

The histochemical staining of hydrogen peroxide (H_2_O_2_) and superoxide radicle (O_2_^−^) was performed according to Muneer et al. (2013).

For H_2_O_2_ localization, fresh leaf samples were immersed in 0.1% solution of 3, 3′-diaminobenzidine (DAB) in Tris-HCl buffer (pH 6.5). The samples were vacuum infiltrated for 5 min and then incubated for 12 h in dark. The leaves were then submerged and bleached in boiling ethanol (95%) to visualize the brown spots and photographed using digital camera.

For O_2_^−^ localization, fresh leaf samples were immersed in a 0.1% solution of nitro blue tetrazolium chloride (NBT), in K-phosphate buffer (pH 6.4), containing 10 mM sodium azide. The remaining steps were carried out as in the case of H_2_O_2_ localization. The formation of blue formazan precipitate was visualized and then photographed using a digital camera.

### Measurement of Tissue Ion Concentration

Around 10 expanded leaves were chosen randomly from each pot. Each treatment group consisted of four replicates. Leaves were dried in a hot air oven for 48 h at 80°C and ground to fine powder. Around 100 mg of leaf samples was acidified with HNO_3_ for 12 h and digested further using a microwave digestion system for elemental analysis. Elemental concentrations were measured using an inductively coupled plasma optical emission spectrometer (PERKIN ELMER OPTIMA 5300 DV ICP-OES; Muneer et al., 2014).

### Histochemical Staining of Silica Body

The histochemical staining of silica bodies in leaf epidermis was carried out according to Yokoyama et al. (2015). Briefly, leaf samples were fixed in FAA solution (formamide, 80% ethanol, and 100% acetic acid) in the ratio 90:5:5 for 24 h in room temperature (RT). This was followed by dehydration treatments with 80, 90, and 100% ethanol for 20 min each at RT. Washing of the dehydrated samples was done with 10, 20, 30, 40, 50, 60, 70, 80, 90, and 100% benzene (in ethanol) for 20 min each at RT. Benzene equilibrated samples were then stained in 0.1% crystal violet lactone solution (in benzene) to visualize the silica bodies in a phase contrast microscope (Model: MT4300L, MEIJI TECHNO CO., LTD., Made in Japan).

## Results

### Si Improved the Formation of Root Nodules in Mung Bean Under Salinity Stress

**Figure 2:**
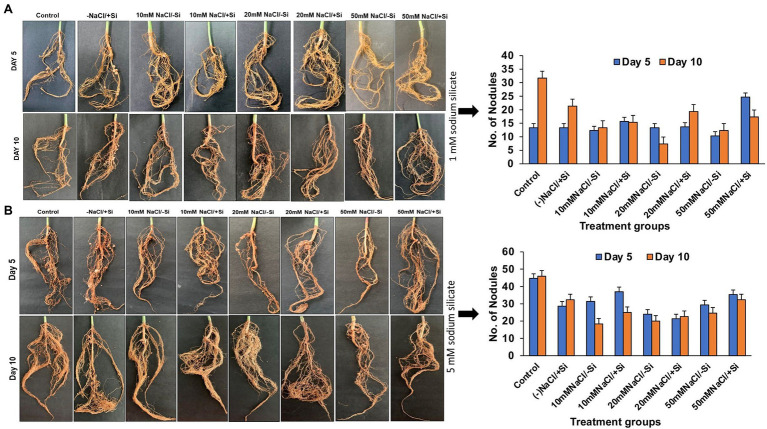
Changes in the formation of root nodules of mung bean (*Vigna radiata*) under **(A)** 1 mM (Si_1_) and **(B)** 5 mM (Si_2_) Si supply and salinity stress after eight treatments: (i) control (T1), (ii) −NaCl+Si (1 mM/5 mM) (T2), (iii) 10 mM NaCl/−Si (T3), (iv) 10 mM NaCl/+ Si (1 mM/5 mM) (T4), (v) 20 mM NaCl/−Si (T5), (vi) 20 mM NaCl/+Si (1 mM/5 mM) (T6), (vii) 50 mM NaCl/−Si (T7), and (viii) 50 mM NaCl/+Si (1 mM/5 mM) (T8) for a period of 5 and 10 days. Vertical bars indicate Mean ± SE of the means for *n* = 4. Means denoted by the different letters are significantly different at *p* ≤ 0.05 according to the Tukey’s studentized range test.

Because legumes have the unique ability to form root nodules for nitrogen fixation, it is thought to be a significant component in legume output. As a result, we looked into whether Si can help with the formation of root nodules under salt stress. According to our observations, the number of nodules formed was relatively limited in the early stages of salt stress (5th day), indicating a more severe loss of root nodules in the later stages of salinity stress (Figures 2A,B). Si_1_ supplementation significantly improved root nodulation on days 5 and 10 of salt stress treatments. The greatest increases in T8 and T6 were 62.07% on day 10 and 58.11% on day 5. Similarly, Si_2_ supplementation increased root nodule formation on days 5 and 10 of salinity stress treatments, with Si_2_ alone showing a significant increase in nodule formation when compared to the control.

### Si Improved the Growth Factors of Mung Bean During Salinity Stress

**Figure 3:**
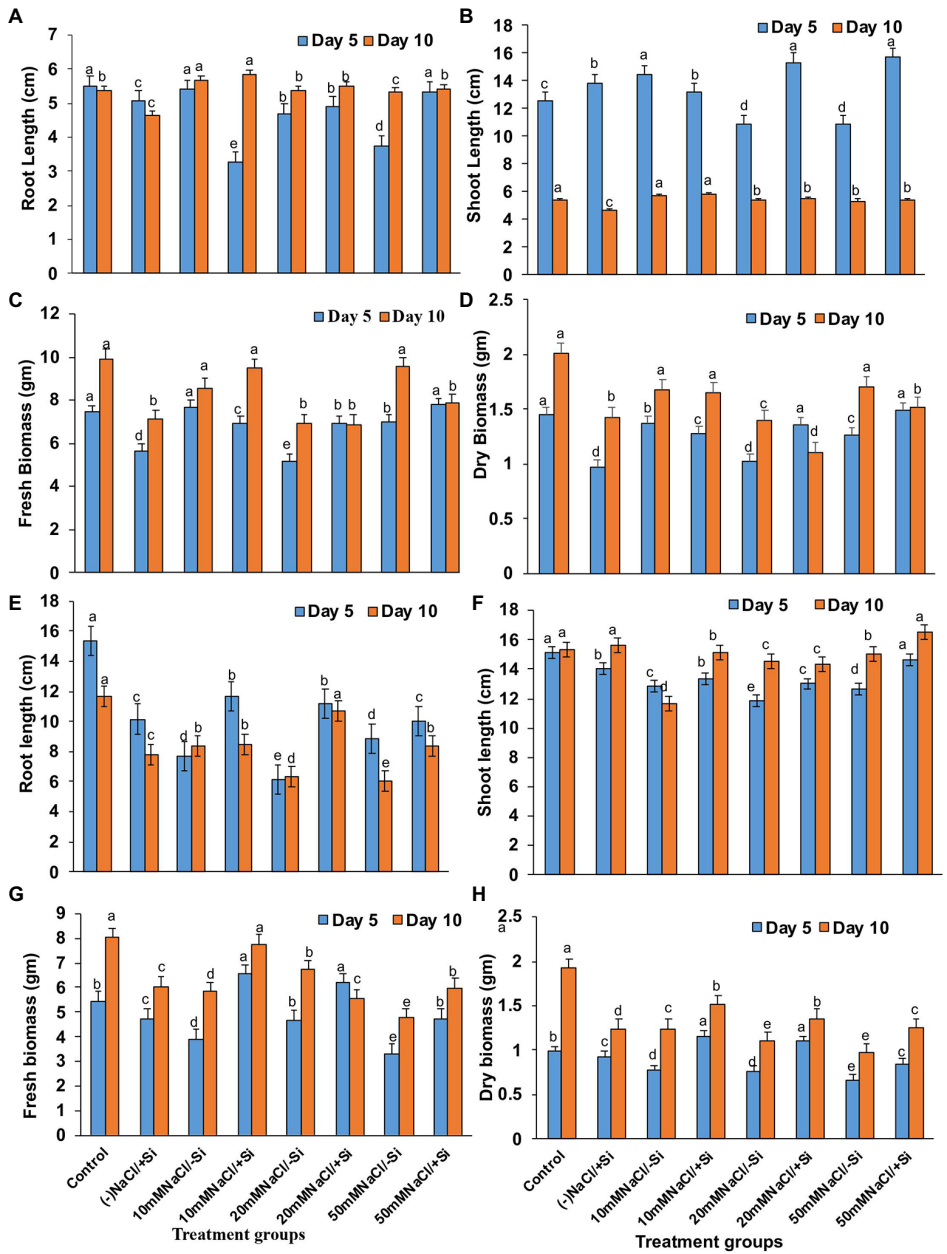
Changes in **(A,E)** root length, **(B,F)** shoot length, **(C,G)** fresh biomass, and **(D,H)** dry biomass of mung bean (*Vigna radiata*) under 1 mM (Si_1_) Si supply and salinity stress after eight treatments: (i) control (T1), (ii) −NaCl+Si (1 mM/5 mM) (T2), (iii) 10 mM NaCl/−Si (T3), (iv) 10 mM NaCl/+Si (1 mM/5 mM) (T4), (v) 20 mM NaCl/−Si (T5), (vi) 20 mM NaCl/+Si (1 mM/5 mM) (T6), (vii) 50 mM NaCl/−Si (T7), and (viii) 50 mM NaCl/+Si (1 mM/5 mM) (T8) for a period of 5 and 10 days. Vertical bars indicate Mean ± SE of the means for *n* = 4. Means denoted by the different letters are significantly different at *p* ≤ 0.05 according to the Tukey’s studentized range test.

On day 5 of treatment, different levels of salinity stress reduced plant root and shoot length (Figures 3A,B). On the fifth day of salinity treatment, however, the application of Si_1_ (1 mM Si) significantly increased root and shoot length. On day 5 of treatment, the increase in root length was found to be around 32% in T8, and the increase in shoot length was found to be 29.33 and 31.13% in T6 and T8, respectively. On the tenth day of salinity treatment, the addition of Si_1_ had no effect on root and shoot length. Similarly, Si_1_ supplementation increased plant fresh and dry biomass on the fifth day of salinity stress but had no effect on biomass on the tenth day of treatment (Figures 3C,D).

However, Si_2_ (5 mM sodium silicate) supplementation resulted in maximum recovery (T4, T6, and T8) across all levels of salinity stress treatments (T3, T5, and T7) on both salinity treatment days (5th and 10th; Figures 3D,E). The root length increased by 44.55 and 40.6% in T6 after 5 and 10 days of silicon treatment, respectively. In the case of shoot length after Si_2_ supplementation, a similar trend was observed. Si_2_ supplementation also resulted in an increase (T4, T6, and T8) in fresh and dry biomass of plants on days 5 and 10 of salinity stress treatment (Figures 3F,G,H). Si supplementation (T2) alone had a positive effect on mung bean growth characteristics during salinity stress.

### Si Improved the Gaseous Exchange Parameters and PSII Quantum Yield During Salinity Stress

**Figure 4:**
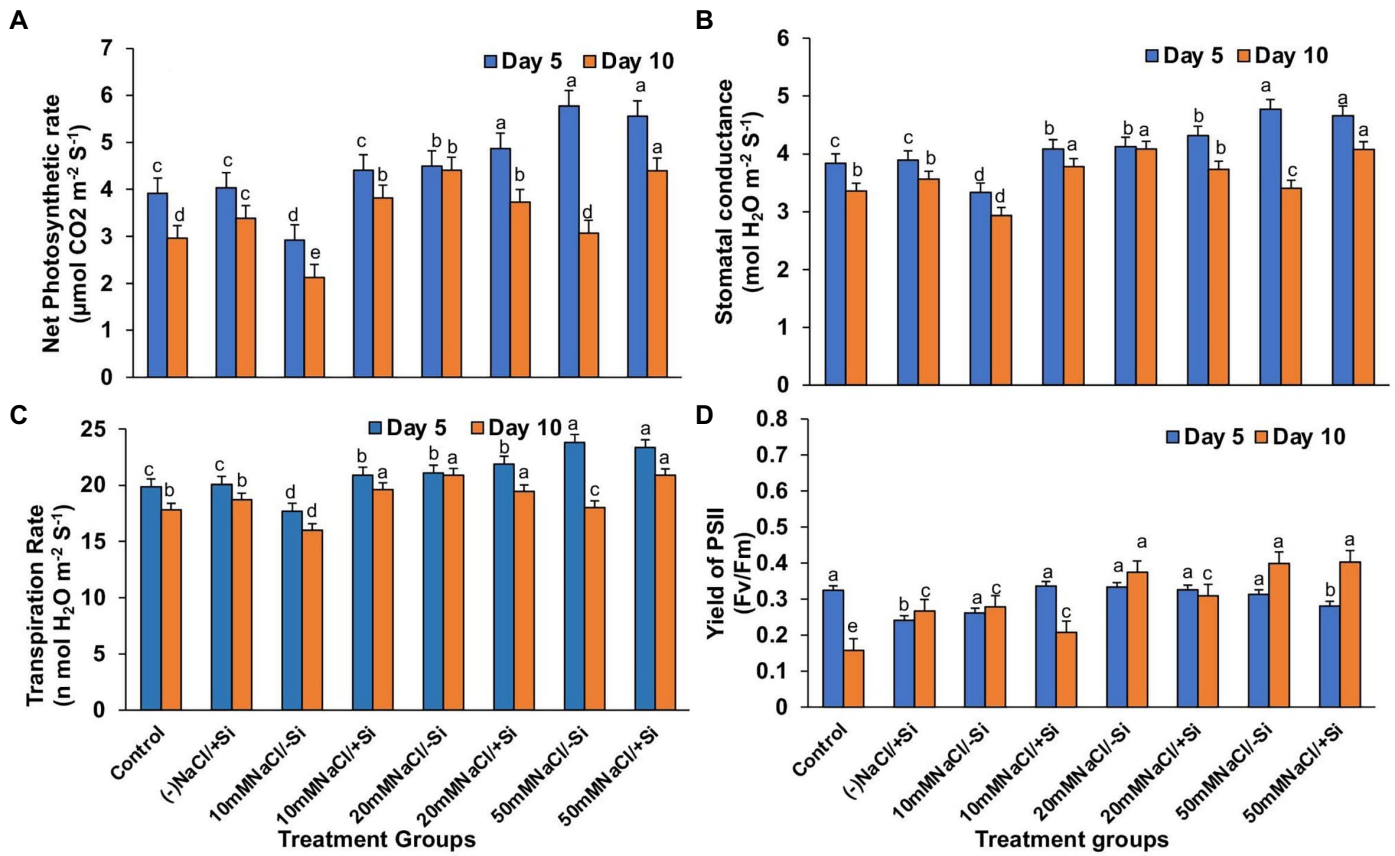
Changes in **(A)** net photosynthesis, **(B)** stomatal conductance, **(C)** transpiration rate, and **(D)** PSII quantum yield of mung bean (*Vigna radiata*) under 1 mM (Si_1_) Si supply and salinity stress after eight treatments: (i) control (T1), (ii) −NaCl+Si (1 mM) (T2), (iii) 10 mM NaCl/−Si (T3), (iv) 10 mM NaCl/+ Si (1 mM) (T4), (v) 20 mM NaCl/−Si (T5), (vi) 20 mM NaCl/+Si (1 mM) (T6), (vii) 50 mM NaCl/−Si (T7), and (viii) 50 mM NaCl/+Si (1 mM) (T8) for a period of 5 and 10 days. Vertical bars indicate Mean ± SE of the means for *n* = 4. Means denoted by the different letters are significantly different at *p* ≤ 0.05 according to the Tukey’s studentized range test.

Except for net photosynthesis rate, which was improved by 34.01 and 7.8% in T4 and T6 on day 5 and by 30.29% in T8 on day 10, the addition of Si_1_ to salinity-treated mung bean did not show much difference in stomatal conductance and transpiration rate (Figures 4A–C). The fluorescence of chlorophyll was also unaffected by Si_1_ addition (Figure 4D).

**Figure 5:**
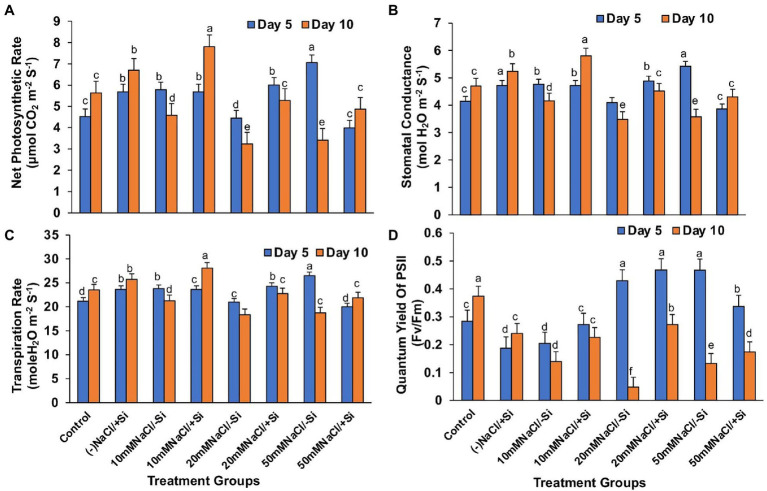
Changes in **(A)** net photosynthesis, **(B)** stomatal conductance, **(C)** transpiration rate, and **(D)** PSII quantum yield of mung bean (*Vigna radiata*) under 5 mM (Si_2_) Si supply and salinity stress after eight treatments: (i) control (T1), (ii) −NaCl+Si (5 mM) (T2), (iii) 10 mM NaCl/−Si (T3), (iv) 10 mM NaCl/+Si (5 mM) (T4), (v) 20 mM NaCl/−Si (T5), (vi) 20 mM NaCl/+Si (5 mM) (T6), (vii) 50 mM NaCl/−Si (T7), and (viii) 50 mM NaCl/+Si (5 mM) (5 mM) (T8) for a period of 5 and 10 days. Vertical bars indicate Mean ± SE of the means for *n* = 4. Means denoted by the different letters are significantly different at *p* ≤ 0.05 according to the Tukey’s studentized range test.

In contrast, applying Si_2_ to mung bean plants under salinity stress increased their net photosynthesis rate by 41.15, 38.82, and 29.97% on day 10 compared to day 5 (Figure 5A). A similar trend was observed in stomatal conductance, which was uniformly improved by Si_2_ on day 10, but there was no significant difference in transpiration rate under salinity stress and/or with Si supplementation (Figures 5B,C). However, Si_2_ addition significantly improved the quantum yield of PSII (*F_v_*/*F_m_*) on day 10 compared to day 5 of salinity stress treatments (Figure 5D).

### Si Increased the Photosynthetic Pigments in Leaves During Salinity Stress

**Figure 6:**
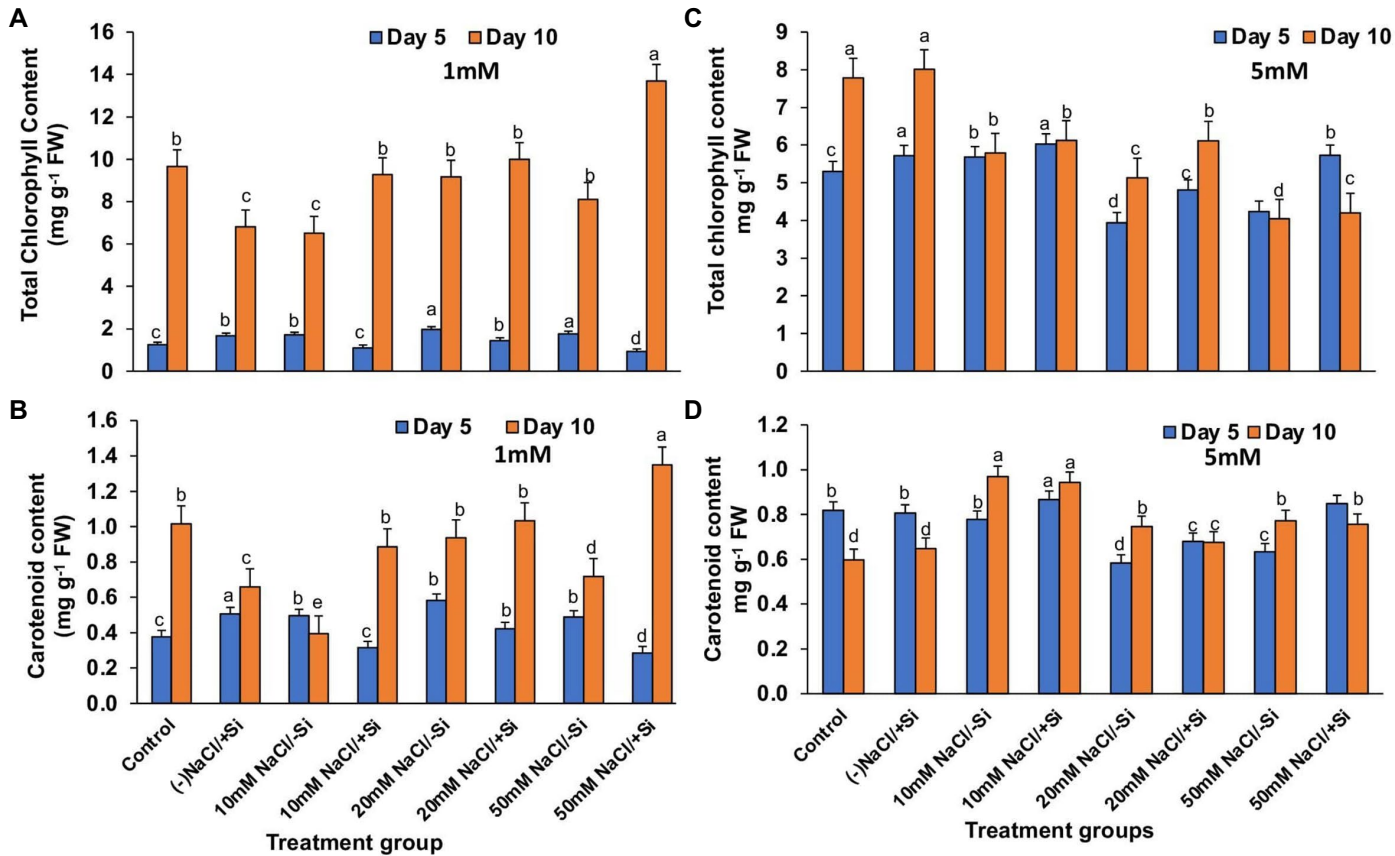
Changes in **(A,C)** total chlorophyll, **(B,D)** carotenoid content of mung bean (*Vigna radiata*) under 1 mM (Si_1_) and 5 mM (Si_2_) Si supply and salinity stress after eight treatments: (i) control (T1), (ii) −NaCl+Si (1 mM/5 mM) (T2), (iii) 10 mM NaCl/−Si (T3), (iv) 10 mM NaCl/+Si (1 mM/5 mM) (T4), (v) 20 mM NaCl/−Si (T5), (vi) 20 mM NaCl/+Si (1 mM/5 mM) (T6), (vii) 50 mM NaCl/−Si (T7), and (viii) 50 mM NaCl/+Si (1 mM/5 mM) (T8) for a period of 5 and 10 days. Vertical bars indicate Mean ± SE of the means for *n* = 4. Means denoted by the different letters are significantly different at *p* ≤ 0.05 according to the Tukey’s studentized range test.

On day 10 of salinity treatment, the chlorophyll content increased by 29.7, 8.40, and 40.71% in T4, T6, and T8, respectively, with Si_1_ supplementation (Figure 6A). The application of Si_2_ had a greater impact on improving pigment content on day 5 of salinity treatment than on day 10, with an increase in chlorophyll content of 5.64, 18.1, and 25.8% in T4, T6, and T8, respectively (Figure 6B). A similar pattern was observed in the increase in carotenoid content during salinity stress as seen in the case of chlorophyll content, with Si_1_ efficiently increasing the carotenoid levels in day 5 and day 10 of treatments and Si_2_ also exerting its beneficial role in improving the carotenoid level more efficiently in day 5 as compared to that in day 10 of treatment (Figures 6C,D).

### Si Improved the Expression of Thylakoid Membrane Proteins Under Salinity Stress

**Figure 7:**
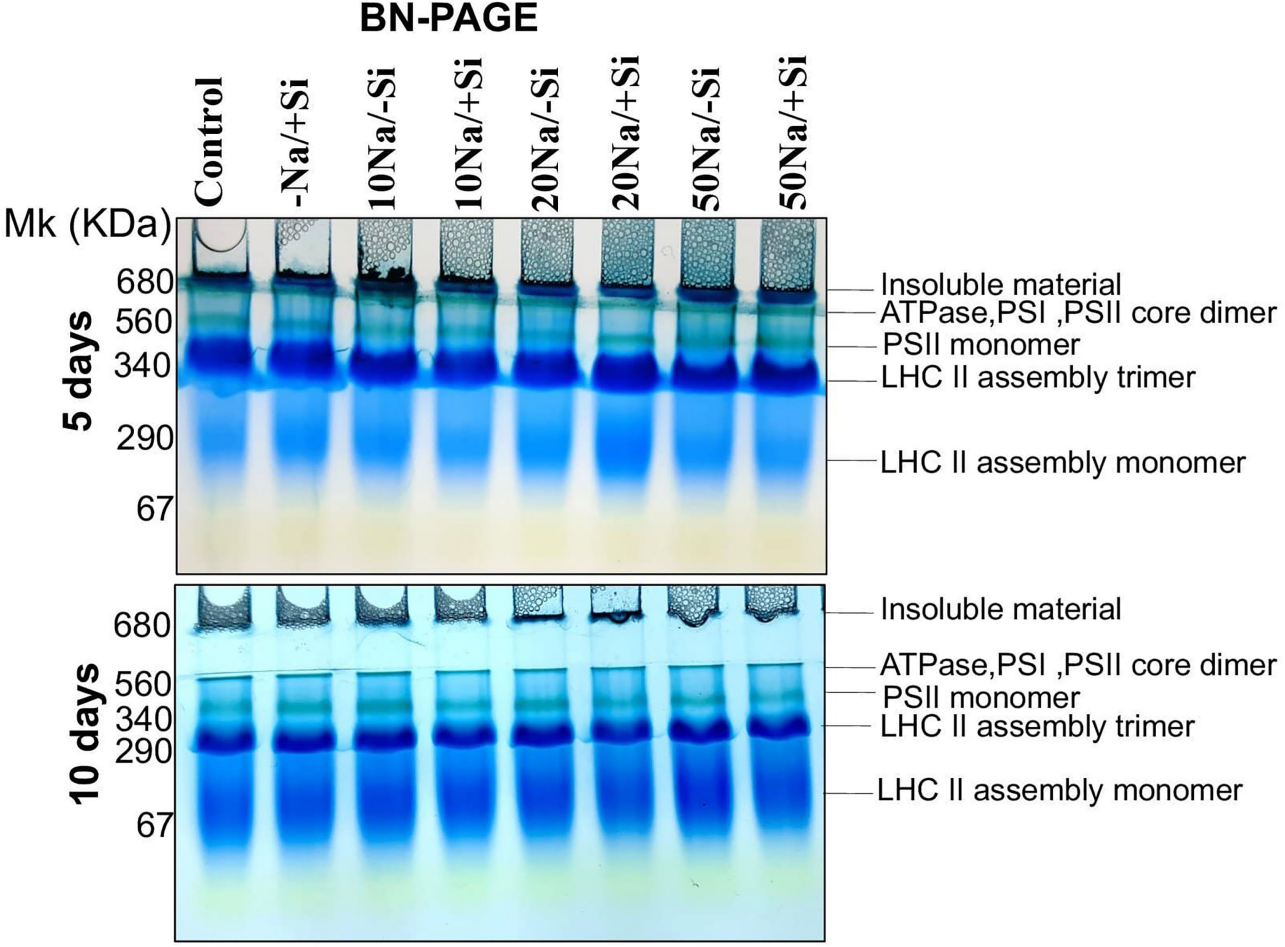
First dimension BN-PAGE of mung bean (*Vigna radiata*) under 5 mM (Si_2_) Si supply and salinity stress after eight treatments: (i) control (T1), (ii) −NaCl+Si (5 mM) (T2), (iii) 10 mM NaCl/−Si (T3), (iv) 10 mM NaCl/+Si (5 mM) (T4), (v) 20 mM NaCl/−Si (T5), (vi) 20 mM NaCl/+Si (5 mM) (T6), (vii) 50 mM NaCl/−Si (T7), and (viii) 50 mM NaCl/+Si (5 mM) (T8) for a period of 5 and 10 days. For first dimension BN-PAGE fresh thylakoid membranes were solubilized in 1% BDM at chlorophyll concentration of 1 μg μl^−1^, and the protein sample was separated by 7–12.5% gradient BN-PAGE.

Since then, Si_2_ has been shown to positively modulate photosynthetic parameters under salinity stress. As a result, we only used Si_2_-treated samples to examine the expression levels of important photosynthetic proteins in order to validate the results obtained from photosynthetic physiological modulations. For salinity-stressed mung bean supplemented with Si_2_, the Blue Native PAGE technique (BN-PAGE) was used (Figure 7). On both days 5 and 10 of treatment, BN-PAGE revealed an increase in the expression levels of PSII monomers in salinity-stressed plants supplemented with Si_2_. Light harvesting complex proteins (LHC II) assembly trimer expression levels were also increased in Si_2_ supplemented groups. Light harvesting complex proteins (LHC II) assembly monomer, ATPase, and PSI core dimer were also found but did not differ significantly between treatment and control groups.

### Si Improved the Leaf Water Status During Salinity Stress

**Figure 8:**
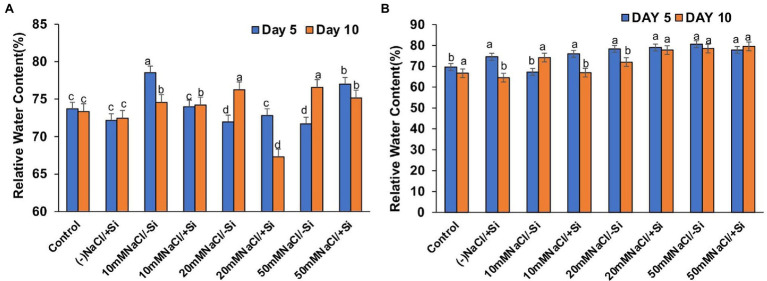
Changes in relative water content of mung bean (*Vigna radiata*) under **(A)** 1 mM (Si_1_), **(B)** 5 mM (Si_2_) Si supply and salinity stress after eight treatments: (i) control (T1), (ii) −NaCl+Si (1 mM/5 mM) (T2), (iii) 10 mM NaCl/−Si (T3), (iv) 10 mM NaCl/+Si (1 mM/5 mM) (T4), (v) 20 mM NaCl/−Si (T5), (vi) 20 mM NaCl/+Si (1 mM/5 mM) (T6), (vii) 50 mM NaCl/−Si (T7), and (viii) 50 mM NaCl/+Si (1 mM/5 mM) (T8) for a period of 5 and 10 days. Vertical bars indicate Mean ± SE of the means for *n* = 4. Means denoted by the different letters are significantly different at *p* ≤ 0.05 according to the Tukey’s studentized range test.

The induction of salinity stress had an effect on the relative water content of leaves (Figures 8A,B). Si supplementation, on the other hand, was found to be effective in restoring the RWC. However, Si_1_ supplementation only reversed the effects of salinity stress in T6 and T8 on the fifth day and had no effect on RWC on the tenth day. On the 5th and 10th days of salinity stress, Si_2_ supplementation was more effective than Si_1_ in restoring water status.

### Si Increased the Levels of Proline Accumulation Under Salinity Stress

**Figure 9:**
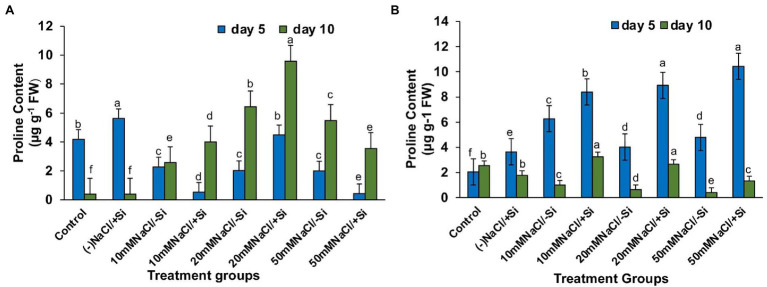
Changes in proline of mung bean (*Vigna radiata*) under **(A)** 1 mM (Si_1_), **(B)** 5 mM (Si_2_) Si supply and salinity stress after eight treatments: (i) control (T1), (ii) −NaCl+Si (1 mM/5 mM) (T2), (iii) 10 mM NaCl/−Si (T3), (iv) 10 mM NaCl/+Si (1 mM/5 mM) (T4), (v) 20 mM NaCl/−Si (T5), (vi) 20 mM NaCl/+Si (1 mM/5 mM) (T6), (vii) 50 mM NaCl/−Si (T7), and (viii) 50 mM NaCl/+Si (1 mM/5 mM) (T8) for a period of 5 and 10 days. Vertical bars indicate Mean ± SE of the means for *n* = 4. Means denoted by the different letters are significantly different at *p* ≤ 0.05 according to the Tukey’s studentized range test.

The accumulation of protective osmolytes such as proline is an important indicator of the “plant stress-response strategy” that confers salinity stress tolerance. On the fifth and tenth days after treatment, we observed a decrease in proline content and an increase in salinity levels (Figures 9A,B). However, Si_2_ supplementation increased proline levels in all plants exposed to salinity stress for 5 and 10 days. After 5 days of salinity stress, the content of proline increased by 69.5, 75.6, and 69.2% in T4, T6, and T8, respectively, and by 25.3, 54.9, and 54.1% in T4, T6, and T8, respectively, after 10 days of salinity stress. Supplementation of Si_1_ was also able to increase the proline content in few treatment groups, but the effect was considered inconsistent as compared to the beneficial effects of Si_2_.

### Si Decreased the Lipid Peroxidation (MDA) Levels During Salinity Stress

**Figure 10:**
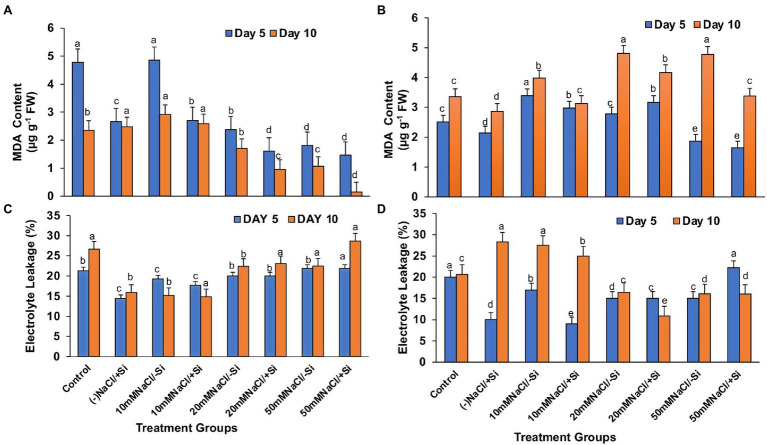
Changes in **(A,B)** malondialdehyde content and **(C,D)** electrolyte leakage of mung bean (*Vigna radiata*) under 1 mM (Si_1_) and 5 mM (Si_2_) Si supply and salinity stress after eight treatments: (i) control (T1), (ii) −NaCl+Si (1 mM/5 mM) (T2), (iii) 10 mM NaCl/−Si (T3), (iv) 10 mM NaCl/+Si (1 mM/5 mM) (T4), (v) 20 mM NaCl/−Si (T5), (vi) 20 mM NaCl/+Si (1 mM/5 mM) (T6), (vii) 50 mM NaCl/−Si (T7), and (viii) 50 mM NaCl/+Si (1 mM/5 mM) (T8) for a period of 5 and 10 days. Vertical bars indicate Mean ± SE of the means for *n* = 4. Means denoted by the different letters are significantly different at *p* ≤ 0.05 according to the Tukey’s studentized range test.

Lipid peroxidation (MDA), expressed as malondialdehyde (MDA) content, was found to increase with levels of salinity (Figures 10A,B). However, supplementation of Si to the salinity-stressed mung bean resulted in a decrease in the lipid peroxidation (MDA) levels, with Si_2_ decreasing the lipid peroxidation (MDA) levels more efficiently up to 27.2, 15.52, and 41.14% with the increasing levels of salinity stress, respectively. Si_2_ was also seen to reduce the lipid peroxidation (MDA) levels under salinity stress, but was not as efficient as Si_1_.

### Si Reduced Electrolyte Leakage During Salinity Stress

Excessive permeability of ions and electrolytes is caused by increased lipid peroxidation (MDA) during salinity stress (Figures 10C,D). As a result, under salinity stress, electrolyte leakage potential increased significantly, which was countered by Si supplementation. When compared to Si_1_, which was partially effective, supplementation with Si_2_ reduced electrolyte leakage potential by 10, 50.64, and 0.625% with increasing levels of salinity stress, respectively.

### Si Alleviated Oxidative Stress in the Form of H_2_O_2_ and O_2_^**−**^ During Salinity Stress

**Figure 11:**
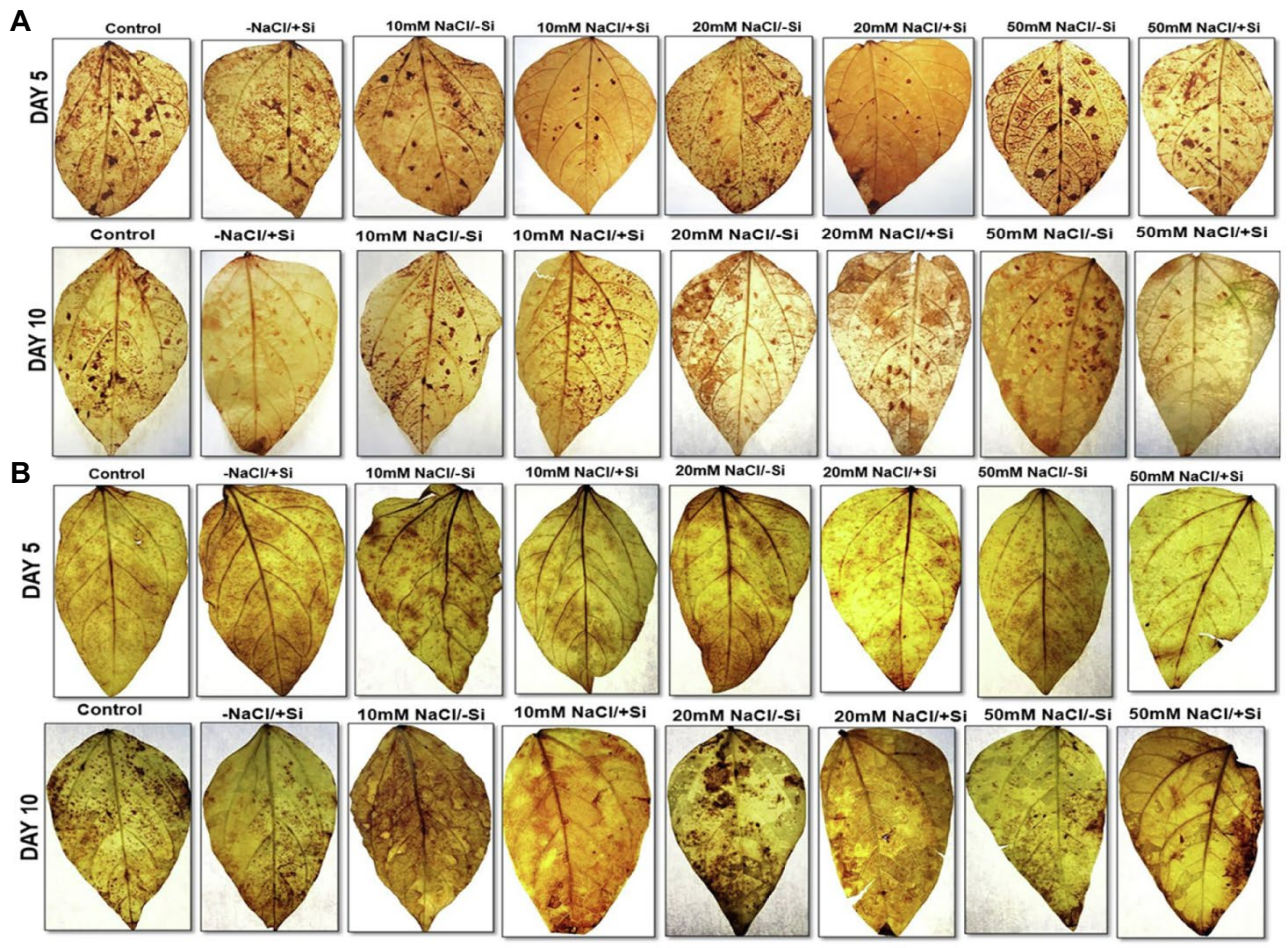
Histochemical localization of H_2_O_2_ in mung bean (*Vigna radiata*) under **(A)** 1 mM (Si_1_) and **(B)** 5 mM (Si_2_) Si supply and salinity stress after eight treatments: (i) control (T1), (ii) −NaCl+Si (1 mM/5 mM) (T2), (iii) 10 mM NaCl/−Si (T3), (iv) 10 mM NaCl/+Si (1 MM/5 mM) (T4), (v) 20 mM NaCl/−Si (T5), (vi) 20 mM NaCl/+Si (1 mM/5 mM) (T6), (vii) 50 mM NaCl/−Si (T7), and (viii) 50 mM NaCl/+Si (1 mM/5 mM) (T8) for a period of 5 and 10 days.

The accumulation of ROS such as hydrogen peroxide and superoxide radical is caused by an increase in lipid peroxidation (MDA) and electrolyte leakage. During salinity stress, the histochemical test revealed that H_2_O_2_ and O_2_^−^ were localized on leaves as dark brown and dark blue spots, respectively. The localization of H_2_O_2_ was observed in all plants after the 5th and 10th days of salinity stress treatments, with an increase in H_2_O_2_ localization with increasing salinity levels (Figures 11A,B). Both Si_1_ and Si_2_ reduced the oxidative stress generated by varying degrees of salt on the 5th and 10th days following treatment, as seen as brown patches, with Si_2_ having the upper hand in the “oxidative stress mitigation” role.

**Figure 12:**
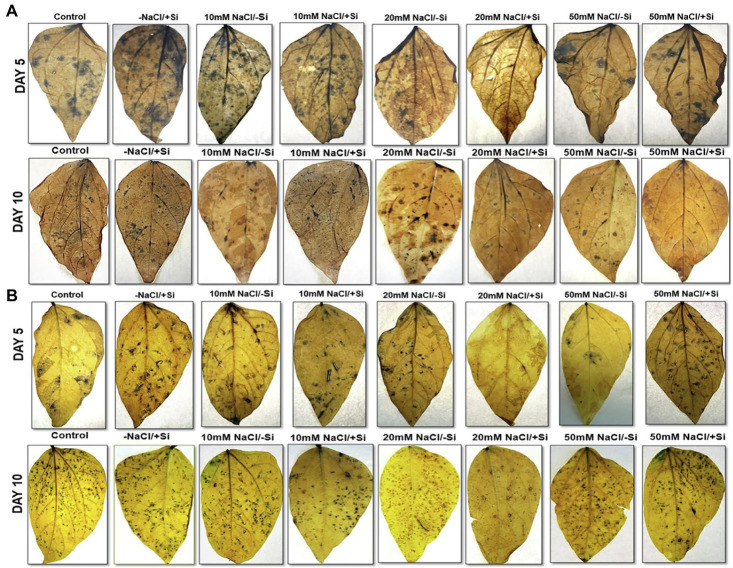
Histochemical localization of O_2_^−^ in mung bean (*Vigna radiata*) under **(A)** 1 mM (Si_1_) and **(B)** 5 mM (Si_2_) Si supply and salinity stress after eight treatments: (i) control (T1), (ii) −NaCl+Si (1 mM/5 mM) (T2), (iii) 10 mM NaCl/−Si (T3), (iv) 10 mM NaCl/+Si (1 mM/5 mM) (T4), (v) 20 mM NaCl/−Si (T5), (vi) 20 mM NaCl/+Si (1 mM/5 mM) (T6), (vii) 50 mM NaCl/−Si (T7), and (viii) 50 mM NaCl/+Si (1 mM/5 mM) (T8) for a period of 5 and 10 days.

The formation of O_2_^−^, which is characterized by a blue spot (due to formazan produced during reaction with NBT), was seen to be more prominent in 10 days compared to the 5th day under salinity stress (Figures 12A,B). However, Si_2_ could scavenge/neutralize the free radical formed, as is evident by the reduction of blue spots in treatment groups T4, T6, and T8 when compared to salinity stress-treated plant groups T3, T5, and T7. Si_1_ was also able to scavenge the O_2_^−^ radical on both 5th and 10th days after treatment, but the level of mitigation was not as significant as seen in Si_1_ supplementation.

### Si Reduced the Na^+^ Uptake and Increased K^+^ Uptake During Salinity Stress

**Figure 13:**
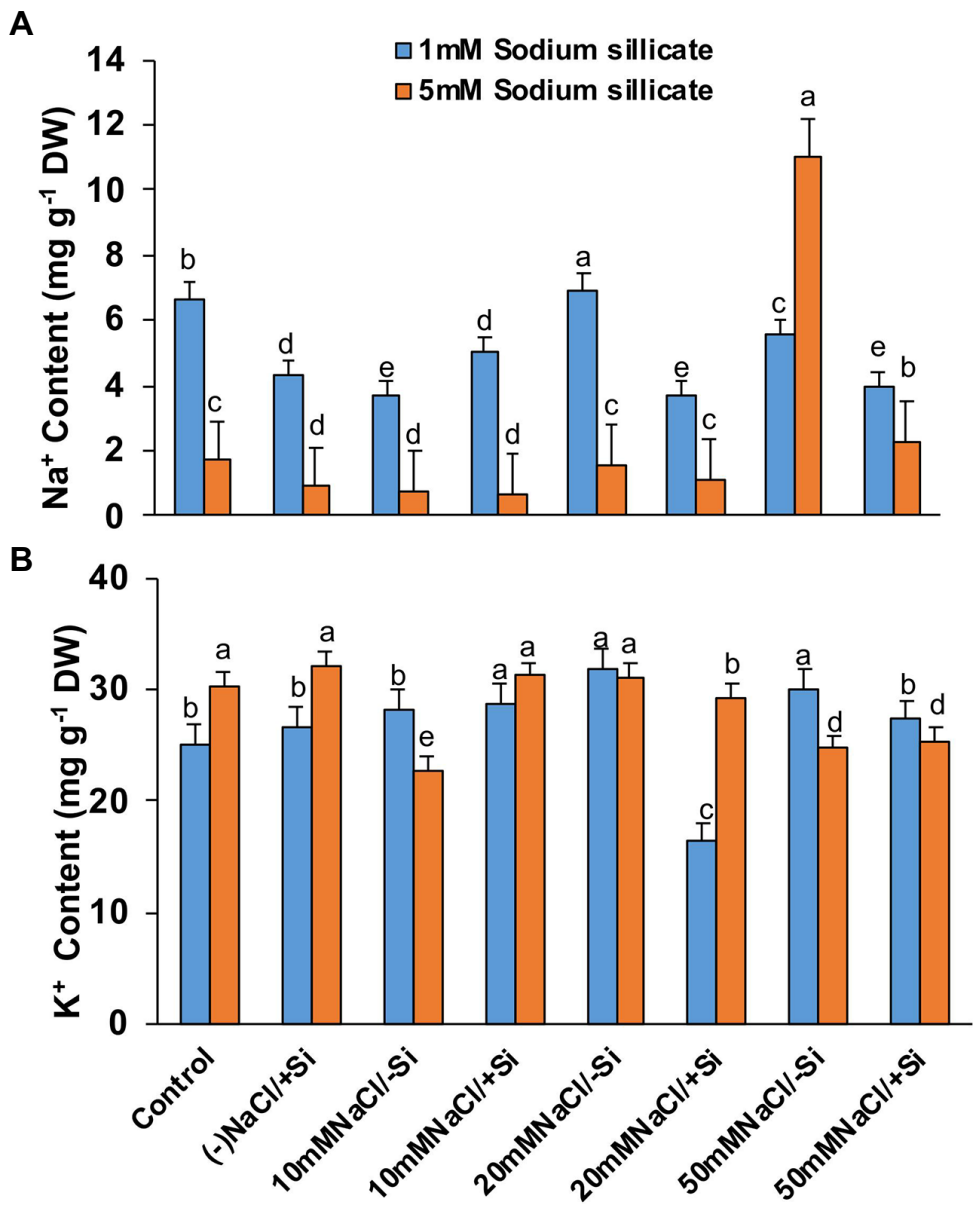
Changes in **(A)** Na^+^ content **(B)** K^+^ of mung bean (*Vigna radiata*) under 1 mM (Si_1_) and 5 mM (Si_2_) Si supply and salinity stress after eight treatments: (i) control (T1), (ii) −NaCl+Si (1 mM/5 mM) (T2), (iii) 10 mM NaCl/−Si (T3), (iv) 10 mM NaCl/+Si (1 mM/5 mM) (T4), (v) 20 mM NaCl/−Si (T5), (vi) 20 mM NaCl/+Si (1 mM/5 mM) (T6), (vii) 50 mM NaCl/−Si (T7), and (viii) 50 mM NaCl/+Si (1 mM/5 mM) (T8) for a period of 10 days. Vertical bars indicate Mean ± SE of the means for *n* = 4. Means denoted by the different letters are significantly different at *p* ≤ 0.05 according to the Tukey’s studentized range test.

The concentration of Na^+^ in salinity-stressed plants increased with increasing levels of salinity, with the highest concentration of Na^+^ observed in T7 (50 mM NaCl) as compared to control (Figures 13A,B). However, Si-mediated obstruction to Na^+^ uptake was reported in the salinity-stressed plants. Si_1_ supplementation decreased the concentration of Na^+^ in treatment groups T6 and T8 by 88 and 42.4%, respectively, whereas the application of Si_2_ decreased the Na^+^ concentration in T4, T6, and T8 evenly, with the greatest decrease in T8 by 393%. The application of Si_1_ and Si_2_ alone also significantly lowers Na^+^ concentration as compared to control. The concentration of K^+^ in salinity-treated plants did not show much difference with or without supplementation with Si_1_ and Si_2_. However, supplementation Si_1_/Si_2_ alone has shown a higher concentration of K^+^ as compared to control.

### Si Uptake and Its Deposition Acts as a Mechanical Barrier During Salinity Stress

**Figure 14:**
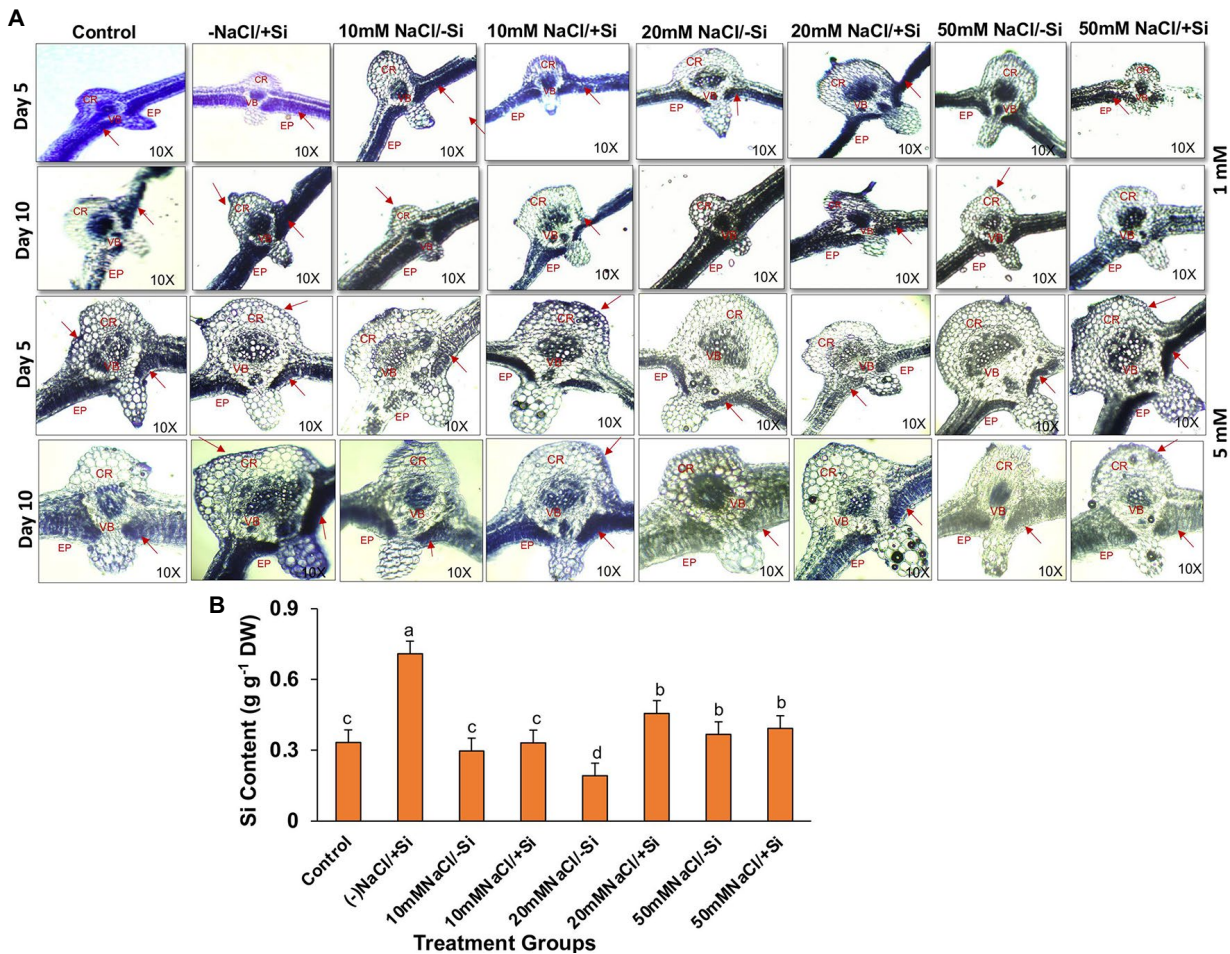
**(A)** Silica deposition in mung bean (*Vigna radiata*) under 1 mM (Si_1_) and 5 mM (Si_2_) Si supply and salinity stress after eight treatments: (i) control (T1), (ii) −NaCl+Si (1 mM/5 mM) (T2), (iii) 10 mM NaCl/−Si (T3), (iv) 10 mM NaCl/+Si (1 mM/5 mM) (T4), (v) 20 mM NaCl/−Si (T5), (vi) 20 mM NaCl/+Si (1 mM/5 mM) (T6), (vii) 50 mM NaCl/−Si (T7), and (viii) 50 mM NaCl/+Si (1 mM/5 mM) (T8) for a period of 5 and 10 days. EP indicates epidermis; CR indicates cortex; VB indicates vascular bundles. **(B)** Silicon concentration in mung bean (*Vigna radiata*) under 5 mM (Si_2_) Si supply and salinity stress after 10 days of treatment. Vertical bars indicate Mean ± SE of the means for *n* = 4. Means denoted by the different letters are significantly different at *p* ≤ 0.05 according to the Tukey’s studentized range test.

Supplementation of Si_1_ to mung bean plants subjected to different levels of salinity could lead to the efficient deposition of silica body in treatment groups T4 and T6 on day 5 of treatment and in treatment groups T6 and T8 on day 10 of salinity treatment (Figure 14A). When Si_1_ alone was applied to mung bean plants, a considerable amount of silica deposition was observed in the leaf epidermis, cortex and vascular bundle. In contrast, Si_2_ supplementation to salinity-stressed plants resulted in an increased uptake of silicon and its subsequent deposition in the epidermis, cortical region and vascular bundle of leaves on both day 5 and day 10 of salinity treatments, as compared to control. When mung bean was subjected to different levels of salinity stress (T3, T5, and T7) alone, the deposition of silica was found to be nearly negligible on both day 5 and day 10 of salinity treatment. However, Si_2_ supplementation alone has shown significant silica deposition in leaves on day 5 and day 10 of salinity treatments. With Si_2_ supplementation showing more silica deposition on day 10 of treatment, similarly, the uptake of Si_2_ on day 10 of treatment was found to be significantly improved in salinity-stressed plants supplemented with Si_2_ (Figure 14B). Silicon uptake was increased by 57.9% in T6 and by 6.6% in T8, respectively. Si_2_ supplementation alone increased the silicon uptake by 53.3% as compared to control.

## Discussion

Most of the Si research has been done on the Poaceae family, thus ignoring the potential role of Si in plant functional groups such as legumes (Fabaceae). Legumes are well known for fixing atmospheric nitrogen in root nodules by forming symbiotic relationship with nitrogen-fixing bacteria (Putra et al., 2020). However, it has been reported that the process of root nodulation in *Lotus japonicus* (López et al., 2006) and *Phaseolus vulgaris* (Faghire et al., 2011) was severely affected by salinity stress. Similarly, in our study, we found that the number of root nodules in mung bean was significantly reduced with increasing levels of salinity. In contrast, on Si supplementation, the nodulation has been found to be drastically improved across all the salinity-treated plant groups, in particular with Si_2_ supply (Figures 2A,B). Our findings are in accordance with Kurdali et al. (2018), who showed that silicon had a more prominent effect on root nodulation during salinity stress in *Sesbania aculeata*. Application of Si to *Glycine max* under stress conditions also increased the nodule numbers when compared to unstressed plants (Steiner et al., 2018). Nelwamondo and Dakora (1999) also reported the beneficial effects of Si supplementation on nodule formation and their function. This increase in nodulation may be attributed to the role of Si in enhancing solute and gas exchange between soil and plant (Putra et al., 2020), structural modulations in nodules by increasing the bacteroids and symbiosomes (Nelwamondo et al., 2001), increased silicification leading to changes in signaling of flavonoids necessary for establishment of symbiosis (Zhang et al., 2013). The results obtained from our study showed that silicon (sodium silicate) particularly 5 mM improved the formation of root nodules in mung bean and depicted that its supplementation can alleviate the negative impact on root nodule formation.

Salinity stress is known for its negative effects on plant growth and performance, where plant biomass remains a crucial indicator for plant stress tolerance (Wei et al., 2017). Our present study suggested that salinity significantly reduced the growth features (root length, shoot length, fresh biomass, and dry biomass) of mung bean, hinting toward inhibition of cell division and inhibition (Figure 3). The decrease in biomass was due to increased NaCl uptake, causing ROS formation, consequently disturbing plasma membrane and ionic imbalance, thus, inhibiting metabolic processes and growth (Mittler et al., 2011). However, the plant growth and biomass were improved by the supplementation of Si. From our results, we observed that Si was able to ameliorate the negative effects of salinity stress on growth, in particular when Si_2_ was supplemented. These findings are in agreement with previous reports of Si-mediated recovery of plant growth in *Cucumis sativus* (Zhu et al., 2020), *Ocimum basilicum* (Farouk et al., 2020), *Sorghum bicolor* (Yin et al., 2013) under salinity stress. The Si-mediated amelioration could be attributed to maintain the integrity of the plasma membrane by efficient Na^+^/K^+^ homeostasis, gene expression stimulation, and enhancement of ROS scavenging machinery (Kim et al., 2014; Daoud et al., 2018).

Salinity stress affects chlorophyll pigment concentrations, lowering photosynthetic rate and reducing plant development. The loss of membrane integrity and the development of enzymes like chlorophyllase, which is proteolytic in nature, cause chlorophyll degradation under stress conditions, may be responsible for the decrease in chlorophyll content (Sabater and Rodriquez, 1978; Tuna et al., 2007). In our study, the gaseous exchange parameters showed a reduction on exposure to salinity stress (Figures 4A–D, 5A–D). Simultaneously, the chlorophyll and carotenoid content were also found to be reduced under salinity stress (Figures 6A–D). In our study, supplementation of Si was prudent in improving the chlorophyll and carotenoid pigment, in particular with Si_2_, which is in line with reports described on *Cucumis sativus* (Feng et al., 2009), *Zea mays* (Khan et al., 2017), *Lactuca sativ*a (Lemos Neto et al., 2020). Si confers protection to chlorophyll due to cell wall deposition of Si resulting in leaf erection leading to higher light interception for photosynthesis (Ma and Yamaji, 2006). The chlorosis and necrosis recorded during salinity stress is stipulated to have reduced the stomatal density, which ultimately affected the stomatal conductance and transpiration rate. However, Si supplementation induced recovery form the detrimental effects on chlorophyll pigment and stomata, leading to the induction of optimal photosynthetic measurements and pigment content. Similarly, Si-mediated improvement of photosynthetic measurements was also reported in *Solanum lycopersicum* (Muneer et al., 2014), *Zinnia elegans* (Manivannan et al., 2015), *Abelmoschus esculentus* (Abbas et al., 2015), which may be attributed to the formation of a double-layered cuticle in leaves that confers mechanical support and maintains photosynthesis in plants (Ma and Yamaji, 2006). The present study also showed a decline in the quantum yield of PSII (*F_v_*/*F_m_*) in mung bean plants during salinity treatments, which is supposedly due to reduction in transfer of energy between antennae and reaction centers (Ghassemi-Golezani and Lotfi, 2015). A reduction in *F_v_*/*F_m_* is an indicator of damage to PSII which affects the photosynthetic rate in turn reducing the plant growth and yield (Rosenqvist and van Kooten 2003). However, Si supplementation was found to be efficient in increasing the *F_v_*/*F_m_* values causing recovery of PSII damage and consequent improvement in chlorophyll fluorescence due to less photoinhibition. The role of Si in maintaining *F_v_*/*F_m_* has also been reported in *Zea Mays* (Khan et al., 2017), *Solanum lycopersicum* (Al-Aghabary et al., 2004), and *Pistacia vera* (Habibi and Hajiboland, 2013). It is hypothesized that the detoxification of ROS mediated by Si leads to an increase in chlorophyll content which leads to the improvement of *F_v_*/*F_m_* (Oukarroum et al., 2015).

The photosynthetic measurements, *viz.* photosynthetic pigments, PSII quantum yield, net photosynthetic rate, transpiration, and stomatal conductance, showed a varied reduction in mung bean under salinity stress which was mitigated by supplementation of Si. From these results, we also depicted that Si_2_ (5 mM sodium silicate) showed more positive effects compared to Si_1_ supply (1 mM Si); thus, we selected the plants for expression analysis of photosynthetic proteins treated with (Si_2_) 5 mM of silicon (sodium silicate) and salinity stress (Figure 7). In our study, we found a reduction in PSI, PSII, monomer and LHC II assembly trimer complex with the exception of LHC-II assembly monomer, under salinity stress. However, supplementation of Si reduced the loss of thylakoid protein complexes. Muneer et al. (2014) reported a similar reduction in thylakoid protein expression in *Solanum lycopersicum* under salinity stress, which was upregulated on Si-supplementation. The damage control of PSI by Si could increase the excitation energy required for NADPH generation from NADP^+^, thus leading to optimal functioning of the photosystem complexes.

The maintenance of metabolic activities during osmotic adjustments under salinity stress is possible when a good water status is maintained in cells and tissues. In our study, leaf water status (RWC) was found to be affected during salinity stress in mung bean (Figures 8A,B). However, application of Si (sodium silicate) was found to restore the RWC in leaves to optimal levels. de Oliveira et al. (2019) also reported that Si (sodium silicate) decreases the water loss by reducing the transpiration rate in Sorghum bicolor. Similarly, Si-mediated osmotic adjustments are touted to be the reason for stress tolerance in *Solanum lycopersicum* (Romero-Aranda et al., 2006), *Sorghum bicolor* (Sonobe et al., 2009), *Zea mays* (Tahir et al., 2012).

During abiotic stress, plants generate and accumulate osmoprotectant as compatible solutes to uphold water for physio-chemical functions in cell (Liang et al., 2018). Proline, a compatible solute, is known to play a crucial role in osmotic adjustments and other protective roles such as protection of protein and membrane structure, scavenging of ROS and cellular homeostasis (Bhaskara et al., 2015). In our study, we found the levels of proline increased with the supplementation of Si to salinity-stressed plants, thus conferring salinity tolerance to mung bean plants (Figure 9). Si-mediated proline accumulation under salinity stress has been reported in *Glycyrrhiza uralensis* (Zhang et al., 2017), *Cucumis sativus* (Zhu et al., 2020) and *Phaseolus vulgaris* (Rady et al., 2019). However, whether an increase or decrease in proline accumulation leads to Si-mediated salinity resistance needs further investigation, as the current knowledge is insufficient.

In plants, reactive oxygen species in low concentrations are needed for the regulation of growth, development and signaling, whereas excessive levels of ROS are synonymous with causing oxidation of membrane lipids, denaturation of proteins, DNA alteration and mutation (Møller et al., 2007; Choudhury et al., 2013). The rate of detoxification is exceeded by the generation of ROS during abiotic stress. Studies have reported that salinity interferes with the process of ROS production and removal, thereby causing more ROS to accumulate, damaging tissue structure and cellular metabolism (Zhu et al., 2015). In our study, salinity stress resulted in an increase in lipid peroxidation (MDA) and electrolyte leakage (Figures 10A–D). Solute leakage is thus associated with a change in membrane lipid composition (Lemos Neto et al., 2020). However, Si supplementation reduces lipid peroxidation (MDA) and electrolyte leakage, which is attributed to the minimum membrane damage as compared to that during salinity stress. Salinity stress also resulted in an oxidative burst, which was seen in the form of H_2_O_2_ and O_2_^−^ significantly localized in the leaves of mung bean during salinity stress (Figures 11A,B, 12A,B). However, on supplementation of Si the oxidative stress was significantly mitigated. Previous research on salinity-stressed plants such as *Capsicum annuum* (Manivannan et al., 2016), *Lycopersicon esculentum* (Muneer and Jeong, 2015), *Lactuca sativa* (Lemos Neto et al., 2020), and *Medicago sativa* (Xiong et al., 2018; Meng et al., 2020) supported our findings. The Si-mediated amelioration of oxidative stress may be due to (a) Si transport and its deposition in the epidermis of cells or in the cortex of vascular tissue, leading to hardening of the cell wall, consequently, preventing the plant from external abiotic stresses (Liang et al., 2003), (b) improved cell turgidity and cell wall extension due to Si polymerization, leading to better transpiration and simultaneous opening and closing of stomata, and (c) activation of antioxidant enzymes to scavenge ROS produced by salinity stress (Tripathi et al., 2017).

Salinity stress negatively affects ion homeostasis in plants, which leads to the accumulation of various toxic ions, leading to ion toxicity and consequently affecting plant growth (Sofy et al., 2020). In our study, salinity stress resulted in an increase in the Na^+^ concentration and simultaneous decrease in K^+^ concentration in mung bean leaves, ultimately affecting the K^+^/Na^+^ ratio (Figures 13A,B). The decrease in K^+^ concentration may be attributed to its physicochemical similarity with Na^+^, leading to competition between Na^+^ and K^+^ ions for binding sites, since the uptake of Na^+^ from roots is facilitated through non-selective K^+^ channels and high affinity K^+^ transporters (Azooz et al., 2015; Miranda et al., 2017; Shahzad et al., 2021). However, we found that Si supplementation resulted in a decrease in Na^+^ content and increased the K^+^ content in mung bean during salinity stress. Reduction of Na^+^ and increment of K^+^ content by Si supplementation were also reported in *Triticum aestivum* (Gong et al., 2005), *Hordeum vulgare* (Liang et al., 2003), *Cucumis sativus* (Wang et al., 2015), *Lycopersicon esculentum* (Muneer and Jeong, 2015), and *Ocimum basilicum* (Farouk et al., 2020). The reduction of Na^+^ content can be attributed to Si deposition in the inner region of the root endodermis, thereby causing obstruction of the apoplastic Na^+^ absorption by roots during salinity stress (Liu et al., 2015). Moreover, our findings on silica deposition in leaf epidermis of mung bean under salinity stress and Si-supplementation further supports this phenomenon. The counteractive uptake of K^+^ against Na^+^ during Si supplementation is due to the activation of plasma membrane H^+^-ATPases of the roots (Karmollachaab and Gharineh, 2015).

The incorporation of Si within plant tissues occurs as amorphous hydrated opaline silica (SiO_2_ × *n*H_2_O), the process of which is widely termed as silicification (Guerriero et al., 2020). Although numerous researches have been carried out to establish the mitigating role of Si during abiotic and biotic stresses, very few reports have been published on imaging silica deposition in plants, and the ones that have been published are from the early 2000s and 2010s. In our study, imaging silica deposition served as a complementary result to the physiological and biochemical data, as it provided spatial information (Figure 14A). We found that silica deposition is more prominent in salinity-treated groups when supplemented with Si. The deposition of silica in leaf improves the leaf rigidity and strength, thereby making the plant more resistant to abiotic stresses in general. Si accumulation varies from species to species with some plants being Si-accumulator and other being Si excluder (Coskun et al., 2018). However, we found significant accumulation of Si in salinity-stressed mung bean which may be due to the deposition of Si on leaf epidermis, indicating that mung bean is a Si accumulator (Figure 14B). The enhanced accumulation of Si in plants under salinity stress have also been reported (Tuna et al., 2007; Muneer et al., 2014; Lemos Neto et al., 2020). The uptake of Si from the soil and its subsequent transport to the leaves and further accumulation of Si in leaf epidermis are the key events which facilitate the overall Si-mediated salinity tress tolerance in mung bean. However, these results are not conclusive and more research into Si transporters at molecular level would enable us to understand the status of mung bean as a Si accumulator or not.

## Conclusion

Pulses are a classic example of the challenge of low returns in a growing market that India’s cash crops are experiencing. Modern markets are all about turning crop shortage into abundance. Hence, the world urgently needs an agricultural approach that is both efficient and climate change resistant. Mung bean is one of the most productive leguminous crops, yet abiotic stressors pose a significant threat to its yield. Due to poor soil and irrigation water quality, salinity stress has long been a key stumbling block for this leguminous crop’s growth and development. Silicon, on the other hand, is regarded as a quasi-essential element for reducing abiotic stressors such as salt. However, there has been a limited number of researches on leguminous crops under salinity stress, with one in particular being a comparison study. In this work, mung bean was investigated under lower (10 mM), intermediate (20 mM), and higher (50 mM) salinity concentrations with two distinct doses of Si, “Si_1_” (1 mM Si) and “Si_2_” (5 mM sodium silicate). Our physio-chemical experiments revealed that Si, in particular the Si_2_ (5 mM sodium silicate) supply, can alleviate salinity stress in mung-bean. Using Si as a supplement, our simulated studies will be able to boost mung bean output regardless of salinity stress.

## Data Availability Statement

The raw data supporting the conclusions of this article will be made available by the authors, without undue reservation.

## Author Contributions

SM conceptualized, supervised, edited, and finalized the manuscript. MA performed the experiments and wrote the manuscript. All authors contributed to the article and approved the submitted manuscript.

## Funding

The research was funded by VIT Seed grant (Project no. SG20210182).

## Conflict of Interest

The authors declare that the research was conducted in the absence of any commercial or financial relationships that could be construed as a potential conflict of interest.

## Publisher’s Note

All claims expressed in this article are solely those of the authors and do not necessarily represent those of their affiliated organizations, or those of the publisher, the editors and the reviewers. Any product that may be evaluated in this article, or claim that may be made by its manufacturer, is not guaranteed or endorsed by the publisher.
